# Defining Health Across the Cancer Continuum

**DOI:** 10.7759/cureus.1029

**Published:** 2017-02-15

**Authors:** Caleb Dulaney, Audrey S Wallace, Ashlyn S Everett, Laura Dover, Andrew McDonald, Lauren Kropp

**Affiliations:** 1 Department of Radiation Oncology, University of Alabama at Birmingham

**Keywords:** health, cancer, survivorship, end-of-life care

## Abstract

Health is not defined by the absence of disease or suffering, but by response to a series of life events that can markedly alter the quality and quantity of life. Patients with cancer experience significant but dynamic physical, psychosocial, and financial challenges. With the increasing number of patients with early stage cancers transitioning to survivorship, there is a critical need to address health promotion and overall well-being. For those with advanced cancer, discussion about prognosis and early integration of palliative care can have a profound impact on the quality of life. Effective communication between healthcare providers and patients is important in aligning treatment recommendations with patient goals and preferences throughout cancer therapy. This review provides a dynamic definition of health and proposes actionable guidelines for health promotion at any point along the cancer continuum: survivorship after early cancer or when goals of care transition to improve quality at the end of life.

## Introduction and background

Health is not a static state, but a series of dynamic transitions during the course of life. Cancer presents several challenges that provide a useful framework for discussion of a dynamic definition of health: a) it affects a diverse population, b) treatment is often expensive, resource intensive, and potentially detrimental to well-being, and c) it is a continuum from early disease with long-lasting effects on overall health to chronic, sometimes life-threatening disease that defines the remainder of a person’s life. The United States has invested substantially in treating this particular disease. However, some argue that a greater portion of resources should be devoted to understanding and improving the quality of life of people with cancer [1]. While the effects of cancer diagnosis and subsequent treatment represent a continuum, it can be divided into two broad categories: early cancer with a focus on health in survivorship and advanced cancer with a focus on the quality of life. The purpose of this review is to formulate a dynamic definition of health and propose actionable guidelines for the promotion of health by discussing the challenges to overall well-being faced by people with cancer.

## Review

### Health after early cancer

Increased cancer awareness and screening have improved diagnosis and outcomes of the most common cancers, such as breast, colorectal, lung, prostate, and skin. It is estimated that the majority of the 1.6 million new cancer cases in 2016 represent purely localized disease [2]. As a result, the nearly 16 million survivors in 2016 are expected to have life expectancies similar to the general population, but with a number of long-term health issues that can impact both survivors and their loved-ones [3]. The American Cancer Society’s Study of Cancer Survivors-II demonstrated that the average survivor has nearly three unmet needs with the top three domains of unmet needs being physical (38.2%), financial (20.3%), and informational (19.5%) [4]. Therefore, the health of cancer survivors faces complex challenges outside the realms of traditional disease-focused healthcare.

Physical and Mental Well-Being

Highly prevalent physical and mental symptoms of cancer survivors include chronic pain, fatigue, anxiety and depression, and sexual dysfunction. These effects can be prolonged as many early cancers incorporate adjuvant therapies that last for many years. In a 2010 Livestrong survey of over 3,000 cancer survivors, 86% reported some physical concern following treatment [5]. The most common concerns are related to energy and concentration, sexual function, neuropathy, and pain. These complex, multifaceted problems are difficult to address and often not assessed by clinicians. Furthermore, loved-ones of cancer survivors may have similar physical and emotional needs that can be overshadowed by those of the survivor. While various oncologic organizations have begun to address concerns of cancer survivors by providing guideline recommendations, the oncology community has a fragmented approach to survivorship management. This may have multiple underlying causes such as ineffective communication, inadequate training of medical professionals, and barriers to multidisciplinary teams. As survivorship care evolves and makes its way into the Centers for Medicare and Medicaid Services (CMS) core quality measures, clinicians accustomed to eradicating disease must become comfortable and confident in managing complex long-term physical and psychosocial health issues. Clinicians should also identify and address these issues in loved-ones of cancer survivors. Such a survivorship framework fits into a definition of health and health promotion in the larger context of human disease.

Health Promotion

Promoting health through diet and exercise following cancer therapy is another important aspect of survivorship. Following treatment for a number of early cancers, the most common causes of death are cardiovascular. Modern cancer therapies also significantly increase a patient’s baseline risk of non-cancer related health problems. Furthermore, diet and exercise interventions following cancer therapy have been shown to reduce mortality and improve quality of life. Fortunately, a number of scientific and professional organizations have published evidence-based guidelines for appropriate diet and exercise recommendations such as the American Cancer Society’s Nutrition and Physical Activity Guidelines [6]. The patient consultation with dietary and nutrition specialists should also be considered for all cancer survivors, especially those with high-risk phenotypes such as cachexia, sarcopenia, obesity, or endocrine dysfunction.

Knowledge and Information

Many patients find the transition from cancer patient to cancer survivor very difficult. The early survivorship period is an important time for patients to focus on personal well-being, but this requires information and communication. While an extensive healthcare support system may exist, many patients are unaware they have available resources and therefore feel great uncertainty during this phase [7]. While many patients feel vulnerable after treatment, the early survivorship period is an opportunity for patient empowerment through knowledge and information. A number of cancer specialty groups have put forward frameworks for cancer survivorship care plans, designed to improve patient certainty and confidence by laying out descriptive recommendations for cancer survivors [8].

Financial Well-Being

Financial security is an important determinant of overall well-being that is often overlooked in discussions of health, resulting in financial hardship in up to 30% of patients [9-10]. Financial well-being is impacted not only by the actual cost of treatment but by the ability to earn income during treatment and in survivorship. Younger age is the primary determinant of the impact of cancer on financial difficulty. Multiple risk factors such as lower income, insurance status, gender, and race are associated with financial hardship rates as high as 49% in younger patients and survivors [9-10]. Overall, 50-80% of people have some change in their employment status, resulting in 60-70% reduction in productivity and doubled rates of financial hardship [9,10-11]. Work related effects from cancer can be long-lasting, with 55% of people worried they may be forced to quit or retire and 32% not seeking career advancement [12]. Women appear to be particularly affected by the loss of productivity and career advancement.

Perhaps most importantly, financial burdens can influence or directly impact physical or mental well-being. There is a dose-response relationship between the extent of financial burden and patient reported pain, physical symptoms, and quality of life that can persist for years after diagnosis [13]. The persistence of financial burden or declining financial status over time in cancer patients is also associated with increased risk of death [11]. Unfortunately, less than 15% of people discuss the costs of cancer care with their providers and when problems arise, the majority feel they have no help largely because of a lack of knowledge about potential resources [12]. Given the effects of the financial burden on overall health and the relatively predictable out-of-pocket costs of standardized therapies, payors and providers should improve communication about expected costs and resources to manage those costs during and after cancer treatment. Providers should also provide more extensive information on how the physical and mental effects of cancer treatment could impact work and productivity.

### Health with advanced cancer

With an estimated 600,000 deaths in 2016, cancer is the leading cause of death in 21 states and the second leading cause in the entire U.S. [2]. However, advanced and metastatic cancer is no longer universally equated with the terminal illness, but a continuum lasting from days to decades. Improvements in cancer treatment have led to a 25% reduction in cancer deaths since 1991 [2]. Many of the treatment regimens for advanced cancer are prolonged and can even last till the remainder of a person’s life. Therefore, the overall well-being of people in this phase of life relies on the discussion of realistic prognosis to guide treatment interventions and adoption of early palliative or supportive care.

Physical and Mental Well-Being

**Figure 1:**
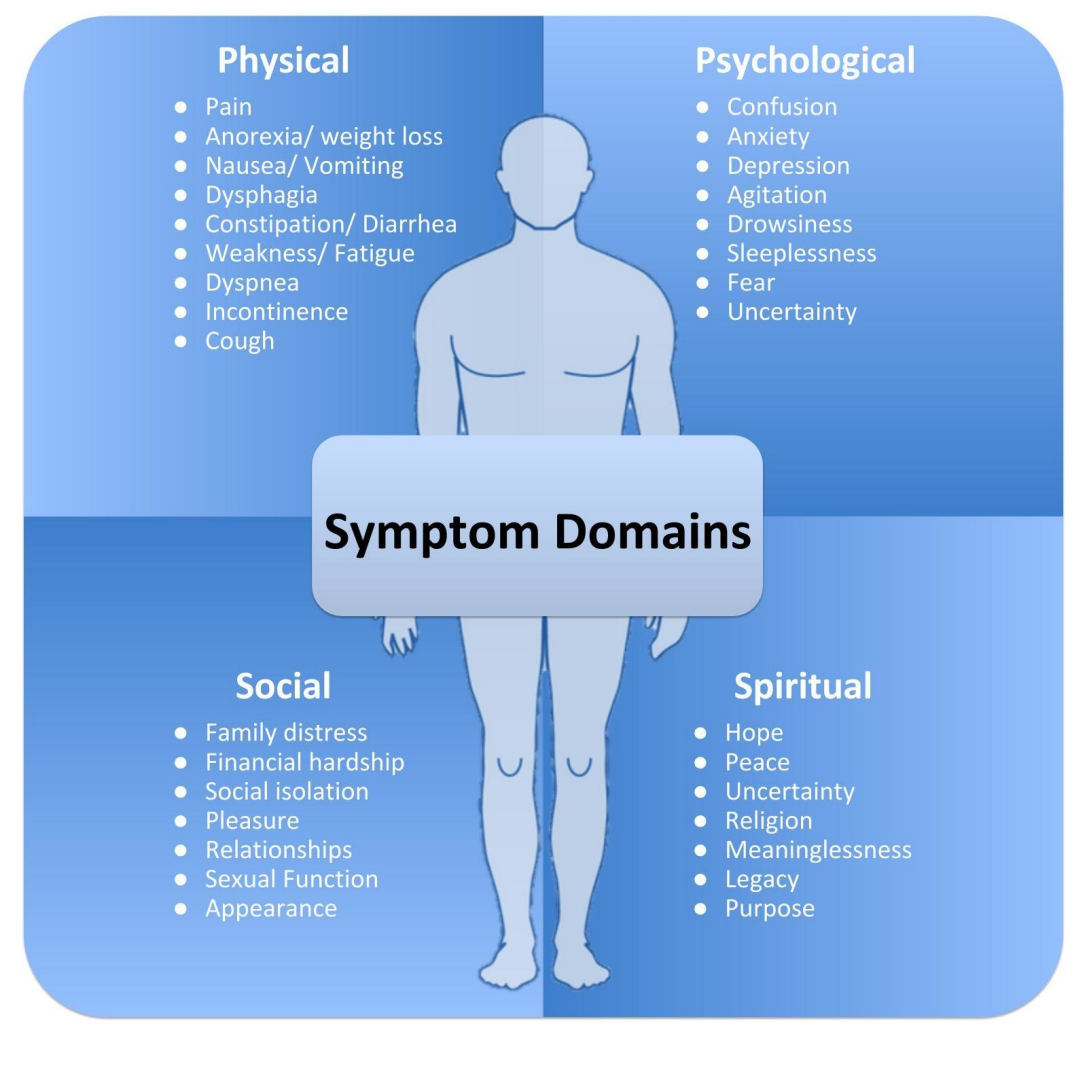
Symptom domains experienced by cancer patients

Patients with advanced cancer, experience many physical, psychological, and social issues that increase in severity towards the end of life [14]. The physical symptoms of advanced cancer often result in significant debilitation, inability to perform basic self-care, and diminished quality of life. In a systematic review, more than 50% of patients experienced five common symptoms: fatigue, weakness, pain, weight loss, and anorexia [15]. Figure 1 presents the complex physical, psychological, social, and spiritual problems faced by patients with advanced cancer [16]. While patients and clinicians often focus primarily on physical symptoms, emphasis should be paid to psychosocial factors as well. A study evaluating distress in advanced cancer showed 16.5%-59.3% of patients experienced some psychological distress, which increased to 70% in the last three months of life [17]. Pain is closely tied to psychosocial symptoms of depression, anxiety, agitation, confusion, sleeplessness, isolation, and feelings of insignificance. Many studies also show that patient's assessment of their symptoms differs from clinician assessment [18]. The issues can also be experienced by loved ones and caregivers but may be ignored or overlooked while caring for the patient. Clinicians must be attuned to patients and caregivers distress level to appropriately identify and treat the symptoms described above. Involvement of multidisciplinary teams of doctors, psychologists and counselors are imperative to relieve patients and caregivers of physical or psychosocial symptoms that can be detrimental to their overall health.

Awareness of Prognosis and Communication with Life-Threatening Cancer

Regardless of where a patient lies on the continuum of advanced cancer, clear communication of prognostic information is important for setting goals and planning treatment when the intent of care is no longer curative. For patients who may live in the range of months, the discussion of imminent death and end-of-life care is a complex and difficult process. Both patients and physicians can overestimate survival by as much as 30%. Patients are often more optimistic than their providers regarding both prognosis and benefits of cancer treatment [19]. This discordance has been shown to improve with conversations about end-of-life care [20]. However, physician concern for causing loss of hope or distress and damaging the patient-physician relationship may prevent open discussions, including the important but very difficult decision to pursue comfort measures. Despite these hesitations, patients consistently report a desire for complete disclosure of prognostic information [21]. It is important for physicians to respect wishes and meet patient needs in the vulnerable period at the end-of-life with open and honest communication.

Early Integration of Palliative Care

End-of-life care for patients with life-limiting cancer is often aggressive and discordant with patient and caregiver preferences mainly due to lack of information regarding prognosis and the effects of treatment [22]. Early integration of palliative and supportive care helps align patient preferences and medical interventions and is associated with improved outcomes for patients and caregivers [23]. As a result, a major component of the American Society of Clinical Oncology’s vision for 2020 is the early incorporation of palliative care into all comprehensive oncology care models [24]. Unfortunately, many cancer patients use palliative care only after failed aggressive care and when death is imminent. Instead, palliative care should start early in the outpatient setting, with the goals-of-care discussion with an established oncologist at a time when the patient can clearly and thoughtfully communicate wishes.

### Valuing health and healthcare in the setting of cancer

**Figure 2:**
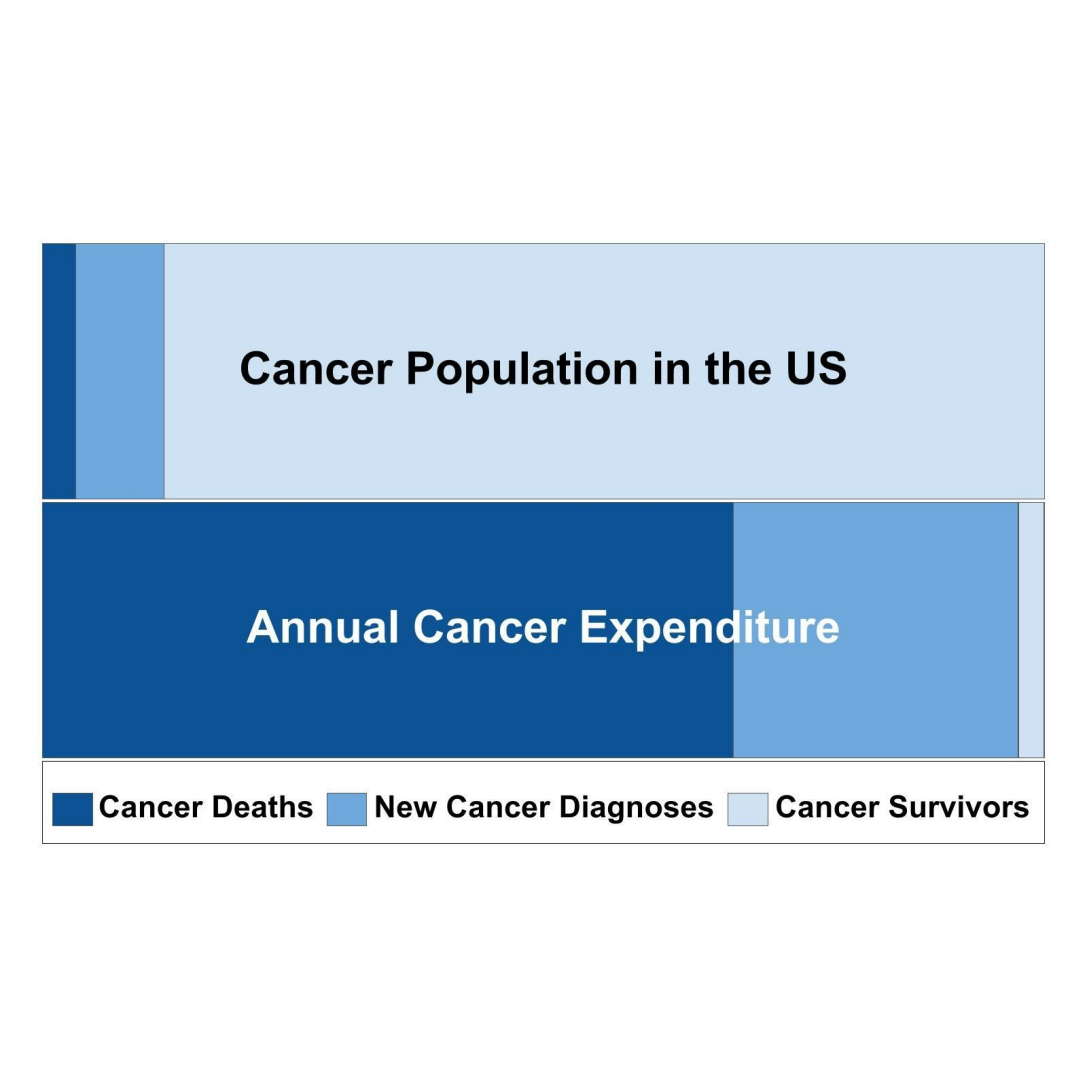
Proportions of the cancer patient population and expenditures by group

Current valuation of health and healthcare largely depends on traditional disease-specific and survival outcomes. However, this approach to healthcare value is changing for two major reasons. First, sophisticated treatments for advanced cancer have resulted in dramatic improvements in traditional outcomes, but at substantial cost. A recent analysis found that Pertuzumab, used to treat HER-2 positive metastatic breast cancer and associated with an improvement in median overall survival of over one year was not cost effective at $472,668 per quality-adjusted-life-year [25]. Figure 2 shows the proportion of patients with new cancer diagnoses, in cancer survivorship and dying from cancer in comparison to the proportion of annual expenditure in 2010 US dollars for each group [26]. As demonstrated in the figure, a small proportion of patients dying from cancer represents the majority of annual expenditure. In contrast, relatively little is spent on the large population of cancer survivors. Second, valuations of cancer treatment have not historically taken into account patient preferences or patient-reported outcomes. Even cost-effectiveness analyses, which account for the economic value of people’s lives do not account for what is most meaningful and fulfilling at the end of a person’s life. The American Society of Clinical Oncology has developed a Value Framework to describe the value of traditional outcomes of cancer therapies in the context of patient-centered toxicity and quality of life outcomes and cost [27]. However, much work needs to be done to determine what outcomes matter most to patients at different stages along the cancer continuum and how those outcomes can be achieved with high-quality and affordable care.

### Actionable guidelines for promoting health

Table 1 presents actionable guideline for promoting the overall health of people with cancer. These guidelines are divided based upon the two cancer populations discussed in this review. The foundation of these guidelines is patient/provider communication and a focus on patient quality of life. For both cancer survivors and those with life-limiting cancer, true value-based care requires a clear definition of what each patient values most and how different treatment approaches support those values. Such an approach still incorporates treatment of disease, but in the larger context of overall health and well-being. Clinicians can empower patients by providing information and knowledge. For survivors, this includes comprehensive survivorship care plans that anticipate common needs and health issues and incorporate concensus guidelines for promoting health. For those with life-limiting cancer, this includes discussions of prognosis, end-of-life symptoms, and clear delineation of both patient and caregiver goals and preferences. The development and implementation of quality measures can provide accountability for the delivery of the best possible care. Finally, including value in the conversation with cancer patients can align their goals and preferences with high-quality cost-effective care.

**Table 1:** Actionable guidelines for promoting health of cancer patients in the setting of value-based care

Cancer survivors	Life-limiting cancer
Survivorship care plans Incorporate guidelines for diet and exercise and expected physical and mental symptoms	Early palliative care Utilize specialized clinics at beginning of the disease process to improve long-term outcomes
Communicate costs Communicate expected costs and resources for managing costs during and after cancer treatment	Communicate prognosis Honestly discuss prognostic information, respecting patient wishes and needs
Survivorship resources Provide easily accessible and understandable resources for survivors	Educate providers Train practitioners and employees to communicate with and provide cancer care in a multi-disciplinary team
Quality metrics for survivorship care Implement metrics improving continuity of care and continued screening for cancer survivors	Quality metrics for end of life care Implement metrics that emphasize improved quality of life aligning with patient goals of care
Value-based care Adopt value-based frameworks to guide treatment decisions that align patient preferences and cost ecost-effectivence-based health care	

## Conclusions

The evolution of patients from diagnosis, treatment, survivorship, and at the end of life requires a full-circle approach where health is defined by what is important and meaningful to patients with cancer. In the setting of cancer and value-based care, health is the response to a series of life events that can markedly alter the quality and quantity of life. Given the increasing number of cancer survivors, as well as those for whom cancer will be a life-limiting event, it is imperative to define the domains that comprise health, the appropriate metrics to evaluate the quality of care provided in achieving health and outcomes that are important determinants of health.
